# Counting the costs of injury and disease to first responders as a result of extreme bushfires

**DOI:** 10.1038/s41598-025-08886-3

**Published:** 2025-07-01

**Authors:** Janneke Berecki-Gisolf, Win Wah, Alex Collie, Deborah C. Glass, Ryan F. Hoy, Malcolm R. Sim, Tim Driscoll, Karen Walker-Bone

**Affiliations:** 1 Monash Centre for Occupational and Environmental Health, School of Public Health and Preventive Medicine, Monash University, Melbourne, VIC Australia; 2 Victorian Injury Surveillance Unit, Monash University Accident Research Centre, Monash University, Victoria, Australia; 3 Healthy Working Lives Research Group, School of Public Health and Preventive Medicine, Monash University, Melbourne, VIC Australia; 4 Department of Respiratory Medicine, Alfred Health, Melbourne, VIC Australia; 5 Sydney School of Public Health, University of Sydney, Sydney, NSW Australia

**Keywords:** Bushfire, Emergency responders, Costs, Workers compensation, Injury/Disease, Health occupations, Epidemiology

## Abstract

Extreme bushfires are devastating and costly and are predicted to increase in frequency. This project investigated emergency responders’ (ER) compensable injury/disease costs associated with extreme bushfire periods compared with the general workforce. Workers’ compensation claims data for Victoria, Australia, were sourced for ER and controls (10% of the general workforce) from January 2005 to April 2021 (encompassing two extreme bushfires). Using generalised linear models, claims from ambulance officers, career firefighters, police, and controls were compared across extreme bushfires, other summers, and all other periods. In total, ER made 749/24,008 (3.1%) claims in extreme bushfire periods, compared to 1254/49,484 (2.5%) in the controls. The study group overall (including both ER and the general workforce control group) experienced significantly higher income compensation costs/claims during extreme bushfire periods, with a 31% increase. ER’ costs/claims were highest for mental illness, burns and cancer. After accounting for bushfire impacts on the general workforce, total claims costs were increased by 67% among firefighters in extreme bushfire periods, largely attributable to fatality payments (other non-medical expenses). These results highlight the need for targeted injury prevention for fatal and non-fatal injuries among ERs and measures that address the broader socio-economic impacts on ERs and the general workforce.

**Supplementary Information:**

The online version contains supplementary material available at 10.1038/s41598-025-08886-3.

## Introduction

Located in south-eastern Australia, the state of Victoria is highly susceptible to bushfires, with a long history of frequent and destructive fire seasons. Bushfires occur commonly in summer months in Victoria, but recent years have been marked by two periods of *extreme bushfire* events. Extreme bushfire events are characterised by their intensity and spread, unpredictable behaviour, devastating impact on human life, property and the environment, and economic and social consequences. These two events have been referred to as the Black Saturday and Black Summer bushfires, which took place in Victoria in 2009 and 2019/20, respectively^1,2^.

First responders dealing with bushfire emergencies are typically firefighters, ambulance personnel, police officers and defence force personnel. Due to their direct and indirect exposure to bushfires and associated environmental, social and health impacts, emergency responders are at increased risk of injury and disease^3–5^. An Australian study reported that emergency responders have higher overall workers’ compensation claim rates than the general working population^6^. The total burden of occupational injury/disease, expressed as time lost from work, was reported to be up to seven-fold increased in first responders compared to the general working population. Previous studies examined the impact of extreme bushfire on occupational injury/disease claims among emergency responders and reported an increased mental health burden associated with extreme bushfire periods^7,8^. First responders had higher overall rates of compensated work-related injury/disease, including mental, musculoskeletal and respiratory conditions compared to the general workforce in both routine^6^ and extreme bushfire conditions^7,8^. These occupational risks are compounded during large-scale disasters, such as bushfires, which expose responders to prolonged physical exertion, high temperatures, traumatic events, and smoke inhalation^3–5^. These health issues have significant consequences not only for the well-being of individual responders but also for workforce retention, service delivery, and compensation systems^6^.

However, less is known about the impact of extreme bushfire periods on compensable injury/disease costs by emergency responders. To anticipate the impact of future extreme bushfire events, a better understanding of the injury/disease cost associated with extreme bushfire events is needed. The *types of costs* (income compensation; hospital; medical service or pharmaceutical costs) specific to claims made in periods of extreme bushfires may also help to inform secondary prevention in emergency responders. The purpose of this study is therefore to determine the compensable injury/disease cost of extreme bushfires among emergency responders.

Although the focus of this study is on emergency responders, extreme bushfire periods are also likely to impact the general workforce due to their effect on air quality and *modus operandi* in the state. Examples of the latter are potential disruptions to power supply; telecommunications outages; road closures and public transport service disruptions; emergency service response delays; and school and childcares closures. In a study in Oregon, USA, increased temperature and wildfire smoke were associated with increased workers’ compensation claims in the overall workforce as well as among outdoor workers^9^.

We, therefore, also sought to test for associations between extreme bushfire periods and claim costs in the general population and to account for this when determining the extreme bushfire burden on emergency responders.

While focused on Victoria, Australia, this study on the impact of extreme bushfires on emergency responders has international relevance, as these events are becoming more common worldwide.

## Methods

### Setting and data sources

This study is set in Victoria, Australia’s second-largest state in terms of population, which was 6.7 million in 2020, 3.4 million of whom were employed^10^. Victoria has a workers’ compensation scheme, which operates as a statutory insurance system, where employers are required to hold a WorkCover insurance policy that covers their employees. The scheme provides compensation and support services to workers with work-related injuries/illnesses. WorkSafe Victoria covers employees for work-related injuries and illnesses, providing compensation for medical treatment, rehabilitation, and loss of income, and does not cover non-work-related injuries and illnesses. WorkSafe Victoria covers work-related mental health conditions, such as those resulting from workplace stress, bullying, or trauma, provided there is a clear connection between the work environment and the condition. To reduce the burden of proof for those in high-stress roles, such as emergency service workers, there is a presumptive acceptance of liability for first responder claims. Certain mental health conditions, such as post-traumatic stress disorder (PTSD), are automatically presumed to be work-related if diagnosed in first responders and certain other emergency workers. For this study, WorkSafe Victoria’s workers’ compensation claims and payments records were accessed. This study was approved by the Monash University Human Research Ethics Committee (Project ID-39153).

### Sample

The study data sample included all claims and claim-related payments for emergency responders from 1 January 2005 onward, based on the date the insurer received the claim. The study end-date was the day of data extraction: 28 April 2023. All payments records were eligible, but claim records dating after 28 April 2021 were excluded, as these did not have two full years of follow-up. Emergency responders were ambulance officers, firefighters, and police officers, selected based on Australian Standard Classification Occupation (ASCO) codes. Defence force claims were not included as these are covered in a separate workers’ compensation scheme and, therefore, were not represented in the WorkSafe Victoria data. Volunteer firefighters are covered by a volunteer firefighter compensation scheme, but these data were not routinely captured in the Victorian workers’ compensation data provided for this research. A control sample was selected as a 10% random sample of all claims and claim-related payments for the entire Victorian workforce during the study period. Emergency responder records were removed from the control sample.

### Outcome

The primary study outcome was claim cost. Claim cost was determined from payments related to each claim and categorised as income compensation, hospital, medical, other and total costs. Costs were indexed to 2023 Australian dollar values. Payments per claim were summarised using a two-year follow-up window from the injury/disease date recorded in the claim: this method was used to enable a fair comparison of claims over time (older claims have had more time to accumulate costs; truncation at two years’ follow-up applied to all included claims eliminated this artefact).

### Variables

Sociodemographic information related to the injured workers was captured as age (ten-year bands), sex, and area of residence. The latter was mapped to regionality according to the Accessibility/Remoteness index^11^ and socio-economic index for area^12^, based on residential postal code. Injury/disease occurrence during extreme bushfire events was determined based on the onset date recorded in the claim: the extreme bushfires took place in Victoria between 7 February and 14 March 2009 (Black Saturday) and 1 November 2019 and 29 February 2020 (Black Summer). All other claims in December, January or February were categorised as summer claims, and the remainder were considered to have occurred off-season (outside of summer and bushfire season). The type of injury/disease was classified according to the Type of Occurrence Classification System (TOOCS)^13^.

### Statistical methods

Claim costs were summarised as mean cost-per-claim and 95% confidence intervals for emergency responders and controls for all claims arising during extreme bushfires and control periods. Cost was broken down by cost category: weekly compensation, hospital, medical (outside hospital), and other. In addition to cost-per-claim calculations, a total sum of costs was calculated for each occupational group and period: this was the cost per claim multiplied by the number of claims in each group and period.

The purpose of the statistical modelling in this study was to determine the effect of extreme bushfire periods on cost-per-claim in emergency responders while taking into account the effect of extreme bushfire periods in the control group (i.e., the general working population). The cost data were non-negative, the distribution was right-skewed, and it contained zeros: to accommodate these attributes, a generalised linear model with Tweedie distribution^14^ and log link was used. The Tweedie distribution belongs to the exponential family, with mean and variance of $\:E\left(Y\right)=\mu\:$ and $\:Var\left(Y\right)={{\upvarphi\:}\mu\:}^{p}$ respectively, where $\:{\upvarphi\:}$ is the dispersion parameter and p is the Tweedie factor: this factor controls the variance of the distribution. Where *p* = 1, the distribution is Poisson; where *p* = 2, the distribution is Gamma, and where 1 < *p* < 2, the distribution is compound Poisson-Gamma. In fitting the data, Tweedie parameters between 1 and 2 were tested, and the optimal factor was chosen based on the lowest corresponding Akaike Information Criterion (AIC). Cost-per-claim was modelled: key independent variables were extreme bushfires periods vs. other periods and occupational groups. Sociodemographic and injury factors were also entered into the model. An interaction effect between extreme bushfire period and occupational group was entered into the model to determine the effect of extreme bushfire periods vs. control period, in emergency responders relative to the effect on the control group. To understand the effect of extreme bushfire periods on each occupational group, a slice statement was used: the results for each cost category are shown in figures. Parameter estimates were exponentiated for ease of interpretation.

## Results

There were 24,008 claims made by emergency responders and 49,484 by the control group during the study period: 749 (3.1%) and 1254 (2.5%) of these, respectively, were made during extreme bushfire periods and 6291 (26.2%) and 11,981 (24.2%), respectively, were made during summer periods.

Comparisons between mean costs per claim in emergency responders and controls, by sociodemographic and injury/disease type, are described in more detail in the Appendix which includes a cost overview table (Table A1). Overall, mean claim costs did not differ between emergency responders and controls but emergency responder claim costs were higher for claims during extreme bushfire periods and claims by older workers. Emergency responder claims costs were lower for many injury/disease types, such as musculoskeletal, fractures, wounds, and circulatory system conditions, but for mental health claims, emergency responder claim costs were higher than those of the control group.

### Claim costs by occupational groups

A breakdown of costs per cost category for each occupational group in extreme bushfire periods vs. all other times is shown in Table 1. Income compensation costs per claim were higher in extreme bushfire periods than in other periods, in all emergency responder groups as well as in controls. Mean hospital costs and medical costs were not statistically significantly different in bushfire period claims compared to other claims in any of the occupational groups. “*Other”* costs, however, were higher in bushfire period claims vs. “other” claims in firefighters. The *other* cost category includes lump sums (such as lump sums paid to dependants after a fatality; common law damages; Sect. 98 impairment benefit); medical and like payments, namely chemist payments; other- paid to other (such as interpreting services); and other- paid to worker (mainly interest paid on weekly payments). For firefighters with extreme bushfire period claims, the majority (72%) of the summed “*othe*r” costs constituted lump sum payments for fatalities.

**Table 1:** Costs* (mean, 95% confidence interval (CI)) for claims related to extreme bushfire periods vs. all other times in the study period.

	Extreme bushfire period claims			Claims in all other periods		
	Mean	95% CI		Mean	95% CI	
Ambulance officers						
Income compensation costs	$20,881	[$15,210,	$26,551]	$13,788	[$12,922,	$14,655]
Hospital costs	$919	[$90,	$1,747]	$1,001	[$870,	$1,131]
Medical costs	$5,484	[$3,756,	$7,211]	$5,355	[$5,095,	$5,614]
Other costs	$10	[$5,	$16]	$423	[$168,	$678]
Total costs	$27,293	[$20,135,	$34,452]	$20,567	[$19,416,	$21,718]
Firefighters						
Income compensation costs	$19,003	[$12,678,	$25,328]	$10,884	[$10,121,	$11,648]
Hospital costs	$1,285	[$628,	$1,942]	$1,482	[$1,308,	$1,656]
Medical costs	$5,885	[$4,448,	$7,322]	$5,103	[$4,512,	$5,695]
Other costs	$10,990	[-$2,701,	$24,681]†	$1,178	[$555,	$1,802]
Total costs	$37,163	[$20,150,	$54,176]	$18,648	[$17,274,	$20,022]
Police officers						
Income compensation costs	$29,923	[$24,081,	$35,766]	$19,838	[$19,090,	$20,586]
Hospital costs	$1,024	[$559,	$1,489]	$1,224	[$1,122,	$1,326]
Medical costs	$6,469	[$5,445,	$7,492]	$6,661	[$6,463,	$6,860]
Other costs	$225	[-$19,	$469]	$540	[$325,	$755]
Total costs	$37,641	[$30,916,	$44,366]	$28,263	[$27,315,	$29,211]
Control sample						
Income compensation costs	$16,074	[$14,283,	$17,865]	$12,503	[$12,254,	$12,753]
Hospital costs	$1,728	[$1,458,	$1,999]	$1,921	[$1,840,	$2,002]
Medical costs	$9,087	[$8,280,	$9,893]	$8,607	[$8,434,	$8,779]
Other costs	$1,509	[$243,	$2,776]	$1,761	[$1,547,	$1,976]
Total costs	$28,398	[$25,527,	$31,269]	$24,792	[$24,305,	$25,279]

*Cost per claim is limited to a two-year follow-up period from the injury/disease onset date. †The confidence interval includes a negative lower limit, indicative of high variability in the data and/or sparsity: the negative lower limit doesn’t reflect negative values but rather the estimate’s wide uncertainty, which expands into the negative range.

### Summed claim costs per cost category

**Fig. 1:**
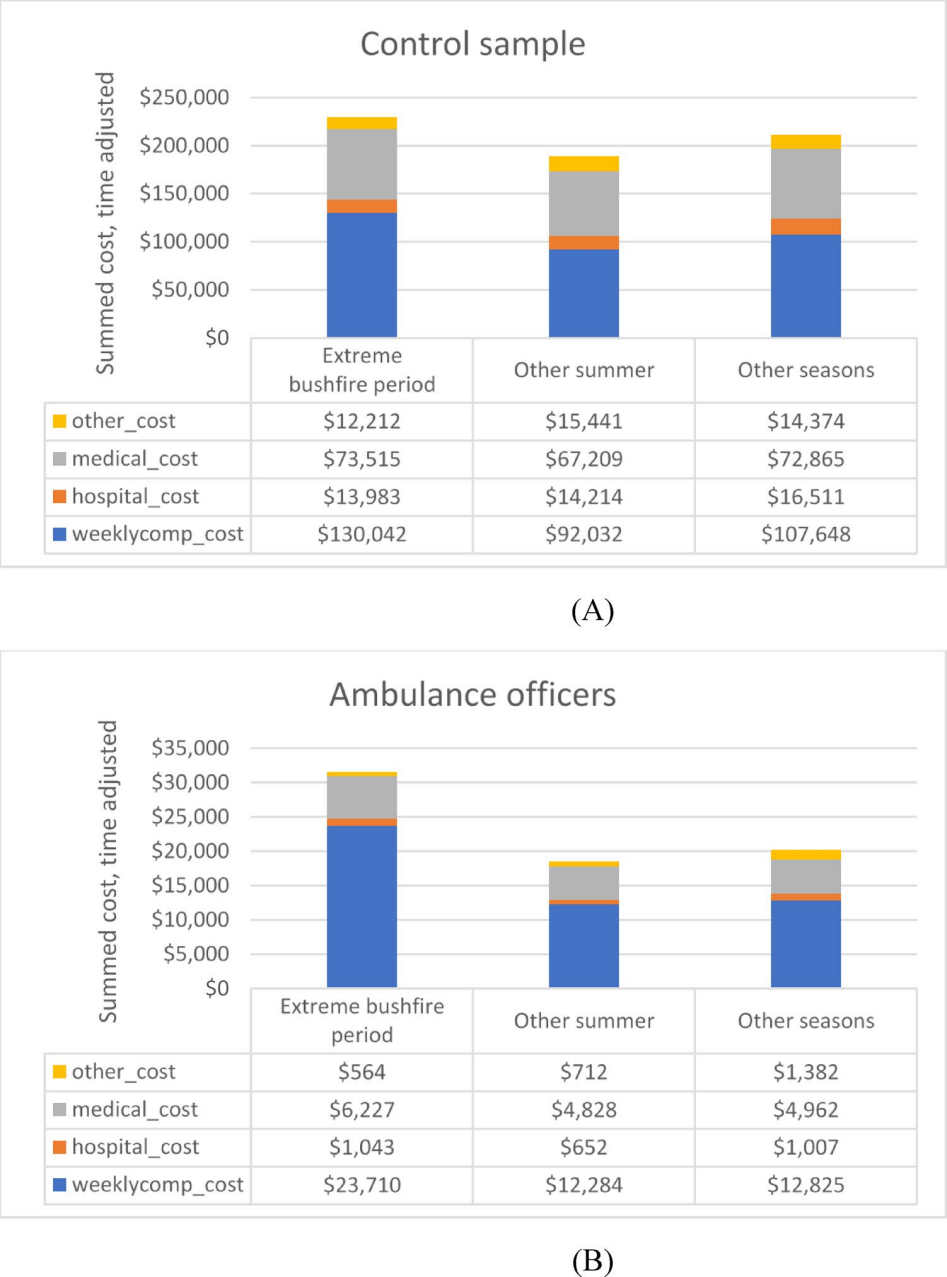

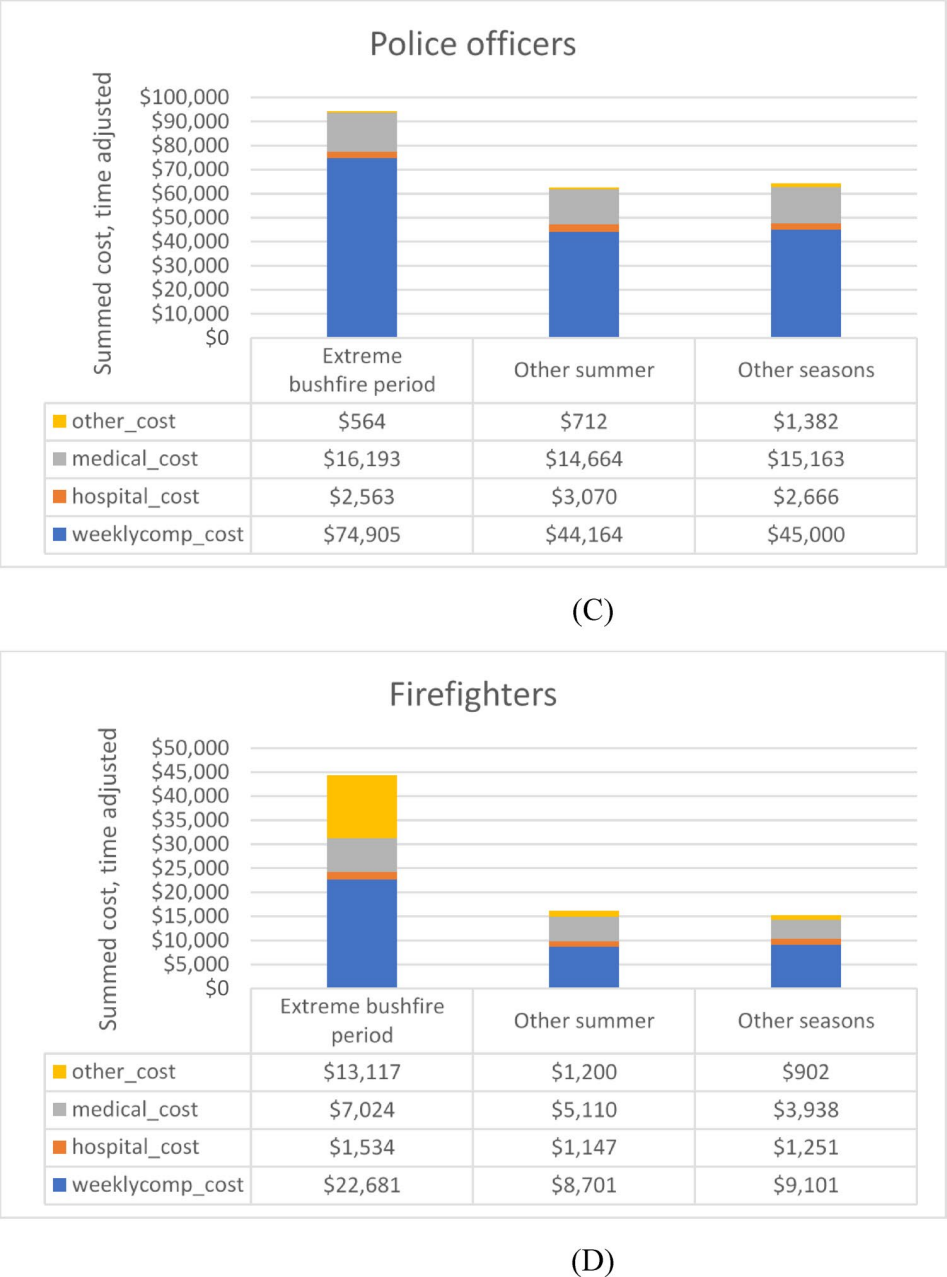
(**A**) Summed claim costs per cost category (weekly compensation costs, hospital costs, medical costs and other costs) in extreme bushfire periods, summer periods and other seasons among control sample. Costs are time adjusted by dividing by the time period duration in days. (**B**). Summed claim costs per cost category (weekly compensation costs, hospital costs, medical costs and other costs) in extreme bushfire periods, summer periods and other seasons among ambulance officers. Costs are time adjusted by dividing by the time period duration in days. (**C**) Summed claim costs per cost category (weekly compensation costs, hospital costs, medical costs and other costs) in extreme bushfire periods, summer periods and other seasons among police officers. Costs are time adjusted by dividing by the time period duration in days. (**D**) Summed claim costs per cost category (weekly compensation costs, hospital costs, medical costs and other costs) in extreme bushfire periods, summer periods and other seasons among firefighters. Costs are time adjusted by dividing by the time period duration in days.

The summed cost of claims made by the emergency responder groups and control sample in the extreme bushfire period, other summer periods (excluding extreme bushfire days) and other seasons, are summarised in Fig. 1. This reflects the cost per claim multiplied by the number of claims, i.e. the cost burden. To control for the differences in duration of these three periods, the summed costs were divided by the number of days in the exposure period, to generate a time-adjusted summed cost. In all groups, summed weekly income compensation costs were higher when they related to extreme bushfire periods compared with other summer or other seasons, whereas for hospital costs and medical costs, there were no pronounced patterns related to these periods. In firefighters, summed *other* costs were considerably higher for extreme bushfire periods than other summer and other seasons: this was mostly attributed to fatality payments.

### Cost modelling

The results of cost modelling are shown in Table 2. Overall, for all claims and the control group, extreme bushfire periods were associated with 31% higher costs for income compensation, 45% lower ‘other costs’ and 15% higher total cost per claim (this is the pattern for all claims, including the control group). Cost-per-claim were lower in all three emergency responder groups than the control group in each of the cost categories and in total cost: ambulance officers, firefighters and police officers were associated with 25%, 34% and 24% lower total claim costs, respectively, than the control group. The interaction effect modelling showed that the extreme bushfire period compared to the control period, in emergency responders *relative to controls*, was associated with 67% higher total claim costs in firefighters (but there was no significant effect in ambulance officers or police officers); this was mainly driven by a stark effect of the extreme bushfire period on *other* costs (mainly payments related to fatalities) in firefighters.

**Table 2:** Results of modelling claim cost* in emergency responders and controls, in the extreme bushfire periods vs. the remainder of the study period†.

		Model 1: Income compensation costs	Model 2: Hospital costs	Model 3: Medical costs	Model 4: Other costs	Model 5: Total costs
Extreme bushfire periods						
*Yes*		1.31 [1.19, 1.46]	0.93 [0.80, 1.08]	1.06 [0.98, 1.15]	0.55 [0.39, 0.79]	1.15 [1.06, 1.26]
*No*		1 [REF]	1 [REF]	1 [REF]	1 [REF]	1 [REF]
*Occupation group*						
*Ambulance officers*		0.90 [0.85, 0.95]	0.73 [0.67, 0.80]	0.59 [0.57, 0.62]	0.35 [0.29, 0.42]	0.75 [0.72, 0.78]
*Firefighters*		0.73 [0.69, 0.78]	0.79 [0.73, 0.86]	0.58 [0.55, 0.60]	0.33 [0.27, 0.41]	0.66 [0.63, 0.69]
*Police officers*		0.88 [0.84, 0.91]	0.81 [0.77, 0.86]	0.66 [0.64, 0.68]	0.31 [0.27, 0.35]	0.76 [0.74, 0.79]
*Control sample*		1 [REF]	1 [REF]	1 [REF]	1 [REF]	1 [REF]
*Bushfire periods x occupation group*						
*Bushfire period*	*Ambulance officers*	1.02 [0.78, 1.35]	1.10 [0.69, 1.77]	0.90 [0.71, 1.14]	0.09 [0.02, 0.37]	1.01 [0.79, 1.29]
*Bushfire period*	*Firefighters*	1.23 [0.93, 1.62]	1.00 [0.65, 1.55]	1.07 [0.86, 1.35]	114.67 [52.36, 251.11]	1.67 [1.32, 2.12]
*Bushfire period*	*Police officers*	1.05 [0.86, 1.27]	1.06 [0.76, 1.47]	0.86 [0.73, 1.01]	1.20 [0.55, 2.62]	1.04 [0.87, 1.24]
*Bushfire period*	*Control sample*	1 [REF]	1 [REF]	1 [REF]	1 [REF]	1 [REF]
*Other period*	*Ambulance officers*					
*Other period*	*Firefighters*					
*Other period*	*Police officers*					
*Other period*	*Control sample*					

*Cost per claim is limited to a two-year follow-up period from the affliction date. †Models 1–5 show the results for each cost category (1–4) and total cost (5); each are adjusted for season,* age group*,* sex*,* socio-economic index for area of residence*,* remoteness and type of injury/disease.*

### Summary of main patterns

**Fig. 2:**
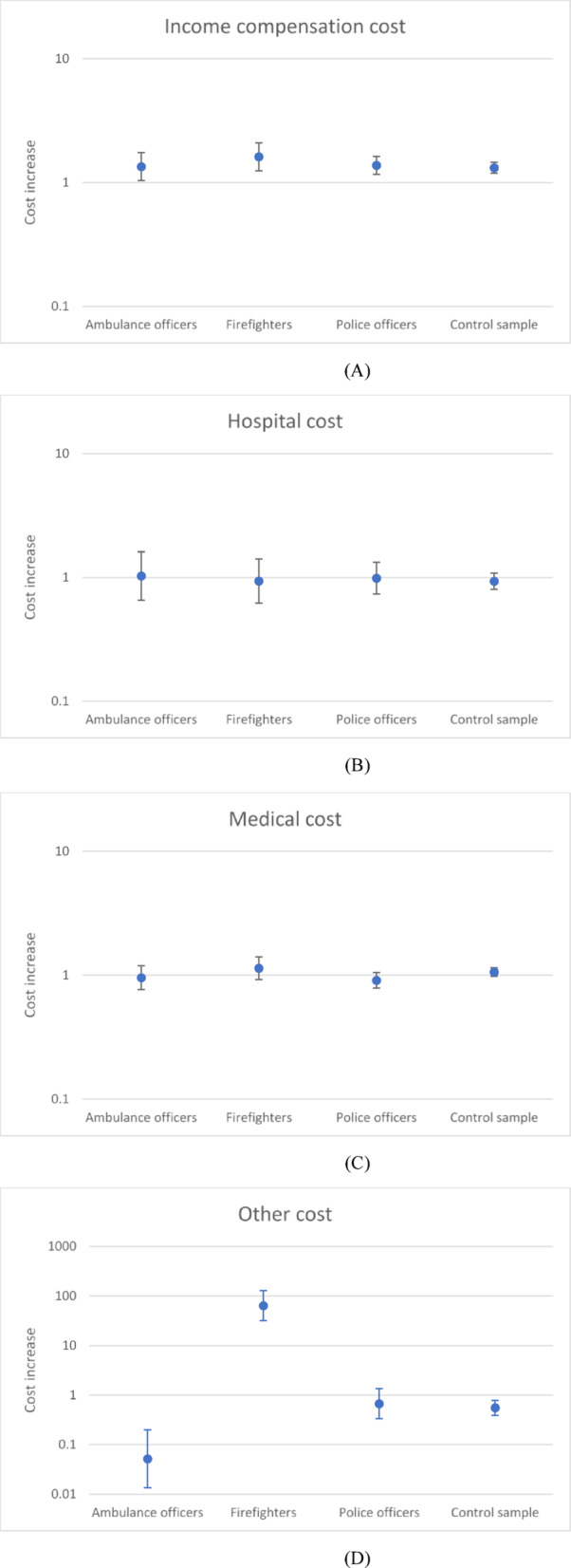

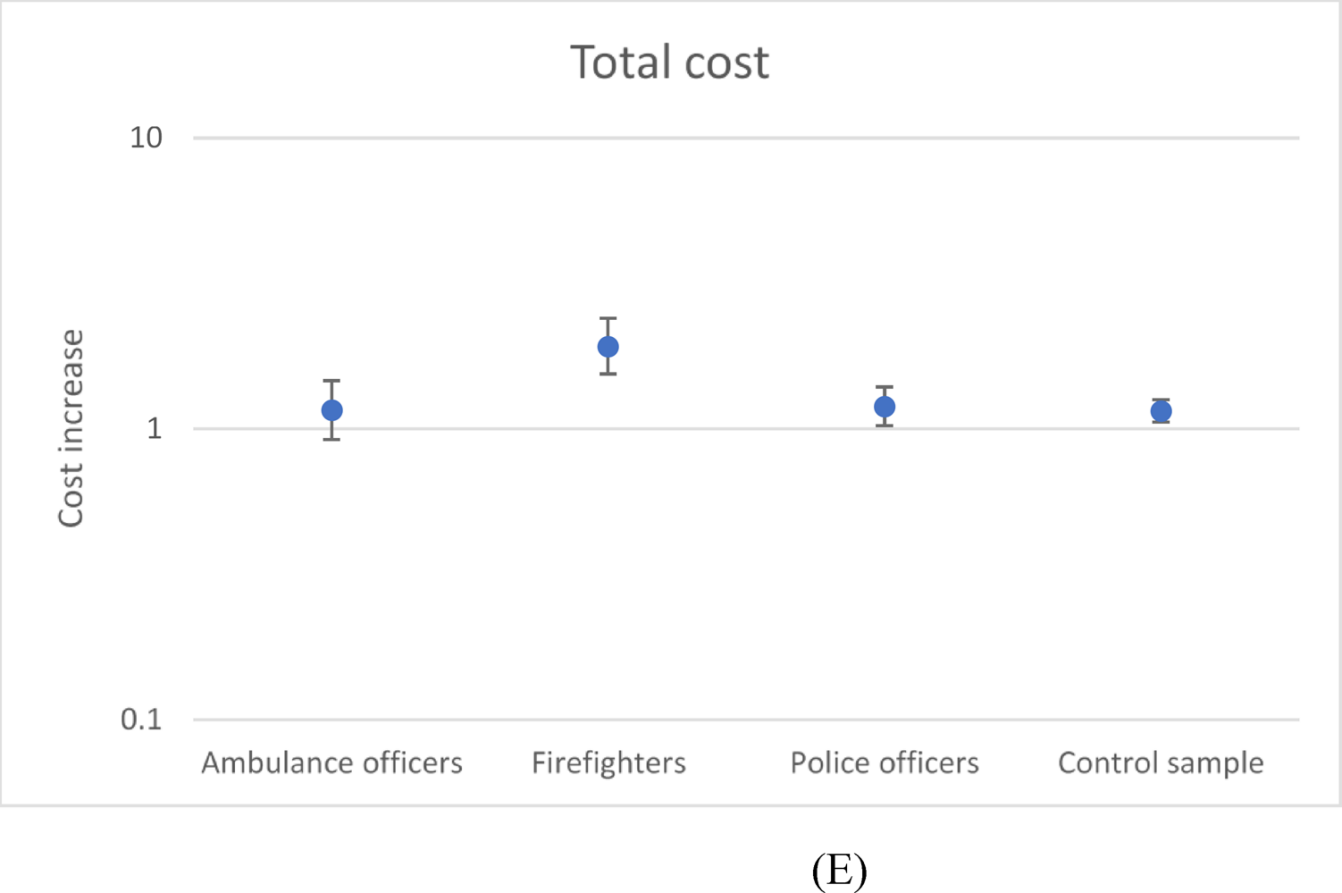
(**A**). Modelled cost increase associated with extreme bushfire periods, compared with the remainder of the study period: Results are shown for income compensation cost type in each of the study groups. Where the 95% confidence interval bars include unity (1), the cost is not statistically significantly different. (**B**). Modelled cost increase associated with extreme bushfire periods, compared with the remainder of the study period: Results are shown for hospital cost type in each of the study groups. Where the 95% confidence interval bars include unity (1), the cost is not statistically significantly different. (**C**). Modelled cost increase associated with extreme bushfire periods, compared with the remainder of the study period: Results are shown for medical cost type in each of the study groups. Where the 95% confidence interval bars include unity (1), the cost is not statistically significantly different. (**D**). Modelled cost increase associated with extreme bushfire periods, compared with the remainder of the study period: Results are shown for other cost type in each of the study groups. Where the 95% confidence interval bars include unity (1), the cost is not statistically significantly different. (**E**). Modelled cost increase associated with extreme bushfire periods, compared with the remainder of the study period: Results are shown for total cost type in each of the study groups. Where the 95% confidence interval bars include unity (1), the cost is not statistically significantly different.

The effect of the extreme bushfire periods on cost per claim in each of the occupational groups, for each cost category, is shown in Fig. 2a-e. Extreme bushfire periods were associated with increased income compensation costs in all groups; hospital and medical costs were unaffected, and other costs were increased in all groups, particularly firefighters. Extreme bushfire periods were associated with increased total costs in all groups except for ambulance officers.

## Discussion

This study of workers’ compensation claim costs during extreme bushfire periods rendered several new insights. Extreme bushfire periods were associated with higher overall occupational injury/disease burden, evidenced from workers’ compensation costs, in emergency responders as well as in other workers (control group). Cost-*per-claim* was also higher in extreme bushfire period claims compared with other claims, in emergency responders as well as controls. Adjusting for the effects of extreme bushfire periods on the control group, an increase in total claim cost associated with extreme bushfire periods was observed in firefighters: this increase was largely driven by *other costs*, which were mainly payments related to fatalities that occurred in the extreme bushfire periods.

### Health and socio-economic impacts of extreme bushfires

The increase in costs for emergency responders, particularly firefighters, was associated with extreme bushfire period claims in our study. aligns with previous studies on occupational health risks faced by emergency responders in both routine^6^ and extreme bushfire conditions^7,8^. as well as among outdoor workers more broadly^15^. In particular, Gray et al. (2017) found substantially elevated injury/disease claim rates among first responders compared to the general workforce, in relation to mental health disorders^6^. The Beyond Blue national study also reported that psychological distress and PTSD prevalence were markedly higher in Australian emergency personnel than in the general population^16^. Our study contributes new evidence by linking these established health burdens to increased economic costs during periods of extreme environmental stress, thereby reinforcing the case for sector-specific mental and physical health interventions. The impact of wildfire-related temperature increases on traumatic injury claim *rates* has been described in outdoor workers in Oregon, USA^9^, but less is known about the impact of (extreme) bushfires on injury/disease claim *costs*.

This study demonstrates an increased cost per claim associated with extreme bushfires, which is not limited to emergency responders but is also observed in the general workforce. This aligns with a study modelling the economic impact of Australia bushfires for 2021 to 2030, which reported that the economic consequences of natural disasters extend beyond emergency responders, affecting the broader workforce with long-term costs for injury and disease management^17^. Our findings further emphasise the need for effective, targeted prevention strategies across occupational sectors.

### Costs of injury/disease claims

Psychological claims tend to result in longer sickness absence than physical claims and are more expensive to treat, with greater costs of rehabilitation and income support^18,19^. Emergency responders were reported to have ten times higher rates of mental health conditions and psychological injuries than the general Australian workforce^6,16^. Ambulance Victoria reported that the average cost of a WorkCover claim in the emergency service grew by almost 25% from 2019 to 20 to 2022-23, which was mainly driven by the relative increases in psychological claims compared to physical claims^19,20^. In our study, the crude overall cost per claim in emergency responders overall was not significantly different from that of the control group in non-bushfire periods. Crude income compensation costs per claim in non-bushfire periods were highest in police officers. While a direct comparison study is not available, these results are consistent with the patterns reported by Gray et al., showing that police had the highest median weeks’ time loss, comparing claims by first responders and controls in Victoria, Australia^6^. It should be noted that in our study, emergency responders had higher proportions of mental health claims than the control group, as also reported in previous studies^7,8^, and these claim types were associated with greater costs. However, the control group had a greater representation of older workers and these were also associated with higher average claim costs. Multivariable modelling showed lower costs per claim in emergency responders compared to controls, overall (the higher claim cost associated with police officers was reversed after adding injury/disease type into the model).

Although not the focus of this study, it was interesting to note that per injury/disease type, the overall cost per claim was sometimes significantly higher (mental disorders) and sometimes lower (fractures, wounds, musculoskeletal injuries, skin and subcutaneous tissue conditions, circulatory system diseases and infections/parasites) in emergency responders compared to controls. This could be related to the broad injury/disease type categories used: for example, circulatory system disease includes hypertension, cerebrovascular disease, ischaemic heart disease, and other specific conditions that can differ greatly in their potential to impact on work ability, requirement for surgery and other health care needs. This noted cost discrepancy could be related to factors such as the relatively fit and healthy emergency responder workforce (healthy selection bias) and potential pressures to return to work in the emergency responder workforce. The difference in cost per claim in emergency responders vs. control group reported in this study, therefore, generates novel research questions and hypotheses, which can be empirically tested in future research on claim outcomes among emergency responders vs. control populations (unrelated to extreme bushfire periods).

### Fatality and injury prevention

The increased workers’ compensation costs associated with extreme bushfire periods in firefighters were related to “*other costs”*: mainly fatality payments. Work-related fatalities are devastating events with enduring impact on survivors. An Australian qualitative study of survivors of traumatic work-related death reported a profound impact on family relationships and long-term behavioural effects on children, as well as health, social and financial ramifications of traumatic work-related death^21^ Our study presents the cost impact on the workers’ compensation scheme, further supporting the need to invest in injury prevention as part of disaster preparedness. Minor, serious and fatal injuries are often presented as the ‘injury pyramid’; however, they are not on a continuum: each requires a dedicated preventive effort. Additional studies comparing causes and risk factors for fatal vs. non-fatal occupational injury/disease associated with extreme bushfires are recommended to help inform specific prevention in each of these domains. Our findings suggest that while fatality-related claims were a significant component of overall cost, tailored prevention programs aimed at reducing both fatal and non-fatal injuries should be a priority for future disaster preparedness planning.

### Broader occupational and economic consequences

The Black Saturday and Black Summer bushfires in Victoria were associated with extreme temperatures, which created the conditions for bushfires, as well as extended periods of poor air quality and pollution levels directly related to the bushfires. It should be noted that the 2019-20 Black Summer Bushfires led to more prolonged smoke exposure over major settlements, compared to the Black Saturday fires^22^. Exposure to these conditions, in varying degrees, was not limited to emergency responders but affected the whole population. The extreme bushfire periods were associated with service disruptions, including power outages, disruption to telecommunications, local road closures, and, in affected areas, closure of schools and public buildings. Overstretching of emergency services responding to the bushfires significantly impacted service availability and response times; in particular, this affected firefighter-, medical- and police services. Disruptions were augmented in the aftermath of the Black Summer bushfires, which coincided with the onset of the Covid-19 pandemic. A qualitative study addressing this compound impact in affected Australian communities mentioned a perceived disruption to the typical disaster recovery processes due to the pandemic; lockdowns, social distancing and fears of contracting Covid-19 reduced community connection and engagement, disrupting the post-bushfire response^23^. The authors emphasised the need for agencies to be responsible for disaster preparedness and coordination of strategies, including cross-jurisdictional collaborations. Such strategies require significant funding. The current research contributes to the evidence of the costs of extreme bushfires and the aftermath, further supporting the justification for investments in secondary and tertiary prevention. Economic evaluations of the burden of bushfires should consider the effects not only on emergency responders but also the general workforce.

## Strengths/limitations

This study has several strengths: it utilises state-wide data, which allows for the calculation of population-based rates and also ensures adequate sample size for robust and sufficiently powered statistical analyses. Using the clearly defined extreme bushfire periods in Victoria provides a novel means of determining the impact on occupational injury and disease costs.

There are, however, also limitations that need to be acknowledged. First, the association of extreme bushfire periods with increased cost of claims that originated in those time frames does not prove that the bushfires caused these cost increases. Other factors may be at play: this study only shows associations, not causal pathways. Second, the Covid-19 pandemic occurred in the period immediately following the Black Summer bushfire periods. Although these events did not coincide, the onset of the Covid-19 pandemic significantly reduced uptake of health services^24–26^. Claims were followed up over a two-year period and therefore, the follow-up period for the Black Saturday bushfires *did* overlap with the Covid-19 pandemic. Claim costs, in particular hospital and medical costs, associated with extreme bushfire periods, may therefore have been greater if it had not been for the occurrence of the Covid-19 pandemic. In other words, the extreme bushfire-associated costs reported in this study may have been subdued due to the effects of the Covid-19 pandemic on health service utilisation (this relates to the second of the two extreme bushfire periods). Third, this study is based on workers’ compensation claim costs. Not all work-related injury and disease will have resulted in a workers’ compensation claim, and when it has, not *all* injury/disease- related costs will have been claimed. The total costs presented in this study are, therefore, likely to be underestimated: however, the patterns, trends and internal comparisons presented in this study are unlikely to be affected by this. And finally, it is important to note that volunteer firefighters, who play an important role in emergency response to bushfires in Victoria, were not included in the analysis: the workers’ compensation data made available for this study did not incorporate data from the volunteer firefighter compensation scheme.

## Conclusion

In conclusion, this study demonstrates that the most recent two extreme bushfire periods in Victoria, Australia, were associated with increased workers’ compensation costs in emergency responders as well as in the workforce overall: this effect was seen in the overall burden as well as cost-per-claim. After adjusting for worker socio-demographic factors and injury type and adjusting for the effect of the bushfires on the general workforce, bushfires were associated with increased cost-per-claim in firefighters (but not in police and ambulance officers): this was mainly linked to fatality payments. These findings support the importance of continued investment in emergency responder injury prevention as a key component of disaster preparedness, in particular, preparedness for extreme bushfires. Further research into the leading causes, circumstances and worker profiles in each of these domains is recommended.

## Electronic supplementary material

Below is the link to the electronic supplementary material.

Supplementary Material 1

## Data Availability

Availability of data and materialsThe claims data on which this study is based cannot be made available by the researchers, but compensation claims data can be requested directly from the data custodians, WorkSafe Victoria, through their data request processes. Conditions apply, including ethical approval. https://www.worksafe.vic.gov.au/research-worksafe. The author, JBG, can be contacted for information on data requests from WorkSafe Victoria if someone wants to request the data from this study. For further information about data access and research at WorkSafe, please email research@worksafe.vic.gov.au.
