# Supplementary material for: Counting the costs of injury and disease to first responders as a result of extreme bushfires
Source: Sci Rep. 2025 Jul 1;15:20769. doi: 10.1038/s41598-025-08886-3 (PMC12215750; doi:10.1038/s41598-025-08886-3)
Supplement: Supplementary file 1 — Supplementary Material 1 [file 41598_2025_8886_MOESM1_ESM.docx]

APPENDIX

Main comparisons of claim costs

The mean costs per claim are summarised in Table A1: overall, claim costs did not differ between emergency responders and controls. In both groups, mean costs were higher for claims that originated in extreme bushfire periods (vs. other time periods) but not for summer claims (vs. other seasons). In both groups, mean costs per claim increased with increasing worker age and mean costs were higher in men than women.

Geographic and socio-economic patterns

Claim costs for workers living in regional Victoria were higher than for workers in major cities: this difference was more pronounced in emergency responders than in the control group. A gradient was observed with decreasing claim cost with increasing socio-economic index for area of residence, in both groups.

Cost variation by injury/disease types

There was a considerable difference in mean claim cost for different injury/disease types. In the emergency responder group, the highest claim costs were observed for mental disorders, followed by burns and neoplasm/cancer. In the control group, the highest claim cost was observed for other injuries, followed by intracranial injuries, and circulatory system disease.

Table A1. Claim cost* (mean, 95% confidence interval (CI)) in emergency responders and the control sample

| *Worker factors* | Emergency responder | | | | Control sample | | | |
| --- | --- | --- | --- | --- | --- | --- | --- | --- |
|  | N Claims (%) | Claim cost (mean [95%CI]) | | | N Claims (%) | Cost (mean [95%CI]) | | |
| Extreme bushfire period |  |  |  |  |  |  |  |  |
| Yes | 749 (3.1%) | $35,091 | [$29,401, | $40,782] | 1254 (2.5%) | $28,398 | [$25,527, | $31,269] |
| No | 23259 (96.9%) | $24,509 | [$23,846, | $25,171] | 48230 (97.5%) | $24,792 | [$24,305, | $25,279] |
| Season |  |  |  |  |  |  |  |  |
| Summer | 6291 (26.2%) | $24,927 | [$23,566, | $26,287] | 11981 (24.2%) | $24,290 | [$23,321, | $25,258] |
| Spring, winter, autumn | 17717 (73.8%) | $24,808 | [$24,045, | $25,571] | 37503 (75.8%) | $25,073 | [$24,520, | $25,626] |
| Age at injury |  |  |  |  |  |  |  |  |
| <25 yrs | 727 (3.0%) | $9,499 | [$8,053, | $10,945] | 4697 (9.5%) | $15,107 | [$13,670, | $16,543] |
| 25-34 yrs | 5132 (21.4%) | $14,359 | [$13,418, | $15,299] | 8868 (17.9%) | $22,440 | [$21,260, | $23,621] |
| 35-44 yrs | 7258 (30.2%) | $21,586 | [$20,511, | $22,661] | 10594 (21.4%) | $26,649 | [$25,610, | $27,687] |
| 45-54 yrs | 7590 (31.6%) | $31,209 | [$29,906, | $32,511] | 13494 (27.3%) | $26,179 | [$25,318, | $27,039] |
| ≥55 yrs | 3301 (13.7%) | $37,017 | [$34,541, | $39,494] | 11831 (23.9%) | $27,538 | [$26,500, | $28,576] |
| Sex |  |  |  |  |  |  |  |  |
| Male | 18266 (76.1%) | $25,384 | [$24,595, | $26,172] | 31965 (64.6%) | $26,130 | [$25,473, | $26,786] |
| Female | 5742 (23.9%) | $23,106 | [$21,896, | $24,316] | 17519 (35.4%) | $22,609 | [$21,976, | $23,243] |
| Regionality* |  |  |  |  |  |  |  |  |
| Major Cities | 15737 (65.5%) | $22,447 | [$21,685, | $23,209] | 36249 (73.3%) | $24,127 | [$23,597, | $24,657] |
| Inner Regional | 6719 (28.0%) | $28,551 | [$27,201, | $29,902] | 11137 (22.5%) | $26,274 | [$25,136, | $27,412] |
| Outer Regional/Remote | 1517 (6.3%) | $33,038 | [$29,539, | $36,536] | 2066 (4.2%) | $30,662 | [$27,839, | $33,485] |
| Socio-economic Index for Area of Residence* |  |  |  |  |  |  |  |  |
| Quintile 1 (lowest) | 2037 (8.5%) | $30,097 | [$27,107, | $33,086] | 8052 (16.3%) | $27,029 | [$25,653, | $28,406] |
| Quintile 2 | 3135 (13.1%) | $29,812 | [$27,816, | $31,807] | 7806 (15.8%) | $26,420 | [$25,069, | $27,770] |
| Quintile 3 | 5240 (21.8%) | $24,751 | [$23,424, | $26,078] | 12236 (24.7%) | $25,273 | [$24,362, | $26,184] |
| Quintile 4 | 6778 (28.2%) | $24,066 | [$22,838, | $25,294] | 11705 (23.7%) | $24,179 | [$23,249, | $25,110] |
| Quintile 5 (highest) | 6767 (28.2%) | $21,768 | [$20,616, | $22,920] | 9604 (19.4%) | $22,221 | [$21,252, | $23,191] |
| Injury/disease type* |  |  |  |  |  |  |  |  |
| Intracranial Injuries | 122 (0.5%) | $21,372 | [$10,388, | $32,357] | 496 (1.0%) | $39,101 | [$29,596, | $48,606] |
| Fractures | 1015 (4.2%) | $17,262 | [$15,166, | $19,357] | 4191 (8.5%) | $33,939 | [$31,966, | $35,912] |
| Wounds | 1527 (6.4%) | $10,005 | [$8,376, | $11,633] | 7002 (14.2%) | $17,766 | [$16,578, | $18,953] |
| Burns | 50 (0.2%) | $41,949 | [$1,790, | $82,108] | 416 (0.8%) | $16,425 | [$11,103, | $21,748] |
| Other injuries | 568 (2.4%) | $13,770 | [$7,593, | $19,947] | 923 (1.9%) | $49,530 | [$38,974, | $60,085] |
| Traumatic joint, ligament, muscle tendon | 4386 (18.3%) | $14,949 | [$14,092, | $15,806] | 7703 (15.6%) | $22,553 | [$21,656, | $23,450] |
| Musculoskeletal system | 8940 (37.2%) | $15,966 | [$15,271, | $16,661] | 17736 (35.8%) | $25,720 | [$25,040, | $26,401] |
| Mental disorders | 5591 (23.3%) | $56,008 | [$54,068, | $57,947] | 4993 (10.1%) | $35,922 | [$34,366, | $37,478] |
| Digestive system | 344 (1.4%) | $16,487 | [$9,286, | $23,687] | 1293 (2.6%) | $13,834 | [$12,698, | $14,970] |
| Skin and subcutaneous tissue | 66 (0.3%) | $3,562 | [$2,454, | $4,670] | 264 (0.5%) | $13,398 | [$10,041, | $16,756] |
| Nervous or sensory organs | 627 (2.6%) | $10,922 | [$9,028, | $12,817] | 3556 (7.2%) | $10,389 | [$9,619, | $11,159] |
| Respiratory system | 109 (0.5%) | $9,218 | [$3,413, | $15,024] | 320 (0.6%) | $10,002 | [$6,799, | $13,204] |
| Circulatory system | 117 (0.5%) | $14,034 | [$7,458, | $20,610] | 207 (0.4%) | $36,496 | [$23,848, | $49,145] |
| Infections and parasites | 91 (0.4%) | $6,732 | [-$1,240, | $14,705] | 66 (0.1%) | $18,208 | [$7,914, | $28,501] |
| Neoplasm Cancer | 254 (1.1%) | $30,648 | [$19,289, | $42,007] | 47 (0.1%) | $15,168 | [-$10,034, | $40,370] |
| Other diseases | 122 (0.5%) | $11,745 | [$4,856, | $18,634] | 99 (0.2%) | $18,336 | [$8,622, | $28,049] |
| Total | 24008 (100.0%) | $24,839 | [$24,173, | $25,505] | 49484 (100.0%) | $24,883 | [$24,403, | $25,363] |

**Cost per claim is limited to a two-year follow-up period from the affliction date.*

*Missing data for regionality: n=67; socio-economic index for area: n=132; affliction type: n=251. A two-year follow-up window was used for determining claim cost.*
